# Social intelligence and pathological gaming: a longitudinal study of the associations among negative emotions, social intelligence, aggression, and pathological gaming in adolescents

**DOI:** 10.3389/fpsyt.2024.1353969

**Published:** 2024-06-06

**Authors:** Sung Je Lee, Eui Jun Jeong, Jae In Choi, Man Su Park

**Affiliations:** ^1^ Department of Digital Culture and Contents, Konkuk University, Seoul, Republic of Korea; ^2^ Department of Media and Communication, Hanyang University, Seoul, Republic of Korea

**Keywords:** pathological gaming, social intelligence, aggression, negative emotions, adolescent gamers, longitudinal study

## Abstract

**Introduction:**

Pathological gaming continues to be highlighted as one of the most critical issues concerning adolescents. Numerous studies have aimed to elucidate the relationships between adolescents' negative emotions (e.g., peer stress, anxiety, loneliness) and social factors (e.g., social skills and relationships) with pathological gaming. Despite the recognition of social intelligence as a crucial factor related to social factors in adolescents, there is a paucity of research examining pathological gaming and social intelligence through longitudinal analyses.

**Method:**

This study focuses on exploring the factors that induce or inhibit pathological gaming among adolescents by analysing three-year longitudinal data from Korean adolescent gamers (N=968). Using a structural equation model, the study examines the relationships between adolescents' negative emotions (e.g., peer stress, anxiety, loneliness), social intelligence, and pathological gaming to elucidate their associations.

**Results:**

The results indicate that negative emotions can potentially reduce levels of social intelligence and increase aggression. Increased aggression, in turn, appears to be associated with higher levels of pathological gaming. Social intelligence was found to impact pathological gaming potentially negatively and may exert a significantly stronger influence on aggression compared to negative emotions.

**Discussion:**

The study's findings suggest that bolstering adolescents' social aptitude and addressing mental health concerns could serve as beneficial interventions in tackling issues associated with excessive media engagement among youth. These findings suggest that, within the context of adolescent pathological gaming, social intelligence could significantly affect aggression and emerge as a key variable that may lead to pathological gaming.

## 1 Introduction

Pathological gaming among adolescents has been reported to impede the attainment of a well-balanced life and pose a threat to the development of social competencies (1, 2). With increasing societal interest in adolescent gamers, extensive research has been conducted on adolescent pathological gaming. Adolescents’ negative emotions can serve as predictive factors for pathological gaming, one of the prominent factors is the negative emotions such as stress and anxiety stemming from the environments in which adolescents find themselves (2–5).

“Pathological gaming” refers to the phenomenon in which users lose control over their gaming activities and excessively immerse themselves in gaming, despite the potential problems such as impaired social relationships, negative mental health issues, and decreased work (3, 6), regardless of these issues. According to prior research, there has been a correlation between symptoms of pathological gaming in adolescent gamers, including depression, anxiety, and aggression, with habitual use being associated with more severe symptoms (7). Likewise, it has also been reported that pathological gaming is associated with aggression and loneliness among adolescents (8).

Follow as several study, social factors have also been reported to have strong relations with pathological gaming. Among social factors, social intelligence has been reported as one of important dispositions related to adolescents’ pathological gaming (9, 10). Adolescents’ social skill or competence is a crucial consideration in the developmental stage where adolescents need to be recognized and accepted in society (11). Previous research adopting such a viewpoint has reported that pathological gaming has negative implications for gamers’ social relationships (12), and social interactions among gamers have emerged as predictive factors for pathological gaming (13). Additionally, a high level of interpersonal stress is also known to be positively associated with pathological gaming (14).

Adolescents’ negative emotions such as anxiety, loneliness, and stress can have adverse effects on their social competence and psychological develo3pment (15–17). Consequently, the deteriorated socio-psychological disposition of adolescents (i.e., low level of social intelligence) can act as a risk factor predicting pathological gaming (18–20). In other words, those in low level of social intelligence could easily suffer from problems in interpersonal relationships, and become vulnerable to pathological gaming (21, 22).

However, despite the potential role of social intelligence in the degree of pathological gaming, there is a notable scarcity of empirical studies analyzing the association of social intelligence with pathological gaming, within the known longitudinal context of adolescence. Therefore, aiming to fill these gaps, we examined an integrated model about the associations among negative emotions (peer stress, anxiety, and loneliness), social intelligence, aggression, and pathological gaming by using three-year longitudinal data from adolescent gamers in South Korea.

## 2 Literature review and hypothesis development

Extensive research has been conducted on adolescents’ pathological gaming, recognized as one of today’s most critical mental health issues (5). Pathological gaming is reported to be associated with the emergence of problematic behaviors, reduced social achievement and failure, and negative outcomes such as interpersonal relationship breakdown (23, 24). This underscores the pressing need for addressing and tackling pathological gaming issues during adolescence. Adolescence is generally recognized as a critical period for laying the foundation for future adulthood in terms of career, social relationships, and life satisfaction. There has been research suggesting that precursors to problematic behaviors in adulthood may stem from issues experienced during adolescence (18, 25).

As reports consistently associate pathological gaming with adverse psychological problems like anxiety, depression, social phobia, and stress, there is a growing demand for a societal response to address pathological gaming (20). According to some studies, there may be an increased risk of gaming-related problems; conversely, individuals may also succeed in integrating gaming into the rest of their lives and experiencing benefits from it (26), However, in the context of the previous discussion, the American Psychiatric Association included the IGD (Internet Gaming Disorder) category in the Diagnostic and Statistical Manual of Mental Disorders (DSM-5), noting it as a section that requires further research. Within this category, the DSM-5 provided nine diagnostic criteria, including preoccupation with Internet gaming, unsuccessful attempts to control Internet gaming use, and continued excessive Internet use despite awareness of negative psychosocial consequences, as well as withdrawal and tolerance (27, 28). Since then, in September 2018, the World Health Organization (WHO) officially adopted the term “game disorder” in the 11th revised edition of the International Classification of Diseases (ICD-11). This classification provided more specific criteria, including a period of at least 12 months of continuous gaming, with weakened control over games, prioritizing games over other life activities, and negative consequences (1).

However, some scholars are concerned that classifying pathological gaming as an mental disease is premature and may inadvertently ignore the positive aspects of video games and precipitate hasty stigmatization effects (29). For instance, some studies highlight the lack of standardized medical diagnostic criteria for pathological gaming and point out that many cases do not consider the potential for comorbid disorders or the influence of social environments, arguing that further academic discussion is necessary (29, 30). A study involving 214 scholars in related fields showed that while 60.8% agreed that pathological gaming could be a mental health issue, still 30.4% of the scholars were skeptical (31). Moreover, only 49.7% of the scholars agreed with the diagnostic criteria for ‘Internet gaming disorder’, and support for ‘Gaming disorder’ was also limited to about 56.5%. This indicates that there is currently no consensus in the academic field on pathological gaming behavior and that contentious points still exist.

Thus, further research is still necessary to clearly understand the pathways leading to pathological gaming, minimize the social harm caused by problematic usage, and provide necessary interventions for those affected. in other words, while video game usage is harmless and enjoyable leisure activity for the majority of players, it can exacerbate serious issues for at least a minority of vulnerable players (12, 32), and can be particularly severe for adolescents who generally have less self-control over leisure activities such as gaming compared to adults (33, 34).

Despite the low prevalence rates and some conceptual controversies, including stigmatization of the gaming industry (29), some scholars have emphasized the need for research to understand the nature and characteristics of pathological gaming. They argue that this understanding is essential not only to provide specialized treatment for affected individuals but also to develop preventive measures (1). Consequently, there is a growing recognition of the necessity to explore factors that either trigger or mitigate pathological gaming (29, 35).

### 2.1 Psychological factors and pathological gaming

Problems in social relationships and the occurrence of negative emotions are among the key characteristics for predicting pathological gaming usage (36). In particular, pathological gaming is known to be triggered through dynamic relationships among various psychological variables, including stress, anxiety, loneliness, aggression, and social intelligence (18–20). However, attempts to verify the complex relationship of each variable and the direction of influence are relatively insufficient, so exploratory research to review the relationship between complex factors and reveal the causal relationship is most necessary.

#### 2.1.1 Peer stress

Peer relationships in adolescence serve as a crucial microsystem for psychological development, particularly playing a pivotal role in the formation of adolescent identity during this developmental stage (37). According to previous research, late childhood and adolescence are known to be periods where fear of physical danger decreases, while social-evaluative fears significantly increase (38). This is because the period of adolescence is marked by an increased importance of peer relationships, along with self-awareness and cognitive maturation. These facts indicate that adolescents may be vulnerable to peer stress arising from social relationships. Peer stress is a negative feeling resulting from a lack of peer recognition (39). Stress occurs when individuals perceive demanding or challenging demands from their environment that they believe exceed their coping abilities. In this context, it can be seen as an individual response to overwhelming (40). High-intensity stress can enhance physiological stimuli that lead individuals to make poor decisions and can also have adverse effects on life balance and mental health (41). Stress can arise not only from shocking or negative events but also when something is important to the person, when the outcome of a specific event is uncertain, or when in situations where others are observing or evaluating (42, 43). Especially, high intensity of peer stress can negatively affect adolescents’ mental health, development, and the growth of their social competencies.

The peer group is another important microsystem that significantly influences adolescent psychological development and behavior (44). Adolescence is a particularly sensitive and highly plastic period, making the potential impact of peer stress-related side effects more pronounced (38, 45). This stage, in particular, witnesses an increased desire for independence from parents and heightened interest in peer relationships, leading to significant changes in social relationships and roles. Consequently, adolescents become more responsive to social stimuli as well (46). For example, adolescents are at a stage where they strive to receive social support, approval, and recognition from their peers (47), and this can lead to a psychological burden to maintain relationships with peers or to avoid negative social evaluations (38). This implies that adolescents are more likely to be sensitive to stress or psychological pressure originating from peer relationships and may also be vulnerable to negative threats due to stress. Therefore For adolescents, the significance of friends and peers implies that these relationships entail substantial amounts of social and emotional interactions and influences in their lives (48). As psychological and social issues become prominent during adolescence, environmental factors within the family and school surroundings play a significant role in stress and mental health (49), an important potential cause of adolescent stress is negative interactions with peers. Many studies emphasize the importance of peers in early adolescence (50): For example, peer stress arising from various social forms such as conflicts with friends, peer exclusion, rejection, or victimization can not only have detrimental effects on the mental health of adolescents, leading to anxiety or depression but also influence aggressive behaviors in interpersonal relationships (50, 51).

Excessive peer stress has been reported to trigger problematic behaviors in adolescents, such as pathological gaming. Adolescents exposed to high levels of stress may engage in game over-involvement as a form of avoidant coping strategy, preferring to immerse themselves in games rather than confronting problems head-on (52, 53). For example, stress and deficiencies in interpersonal relationships can influence escapist immersion in online games (54), and the lack of real-life success experiences and the psychological burden from minor achievements can contribute to over-involvement in games that offer easy rewards (53, 55). Additionally, other studies have shown that groups with pathological gaming have significantly higher scores in interpersonal stress than those without (14). In this context, it can be said that adolescents who fail to form positive interpersonal relationships or experience stress as a result are more likely to exhibit maladaptive behaviors such as pathological gaming.

#### 2.1.2 Anxiety and loneliness

Anxiety is known as a psychological state characterized by persistent and excessive worry that interferes with daily activities, accompanied by physiological tension such as palpitations or trembling and negative emotions (56, 57). Anxiety during childhood and adolescence, in particular, is known to be closely associated with mental health issues such as depression. Additionally, it can hinder the development of social skills and lead to problems such as impairment in peer relationships (58, 59). For instance, a study involving 1,305 high school students revealed that adolescents with high scores in social anxiety were more likely to have a negative self-image (60).

Anxiety is known to be closely related to pathological gaming. Individuals with high social anxiety may experience stress and distress in face-to-face social interactions but can find psychological comfort in online gaming environments where they can hide themselves (61). In particular, the structural characteristics of games, such as achievement, anonymity, convenient social relationship building, and variability, can help people forget real-life problems and offer attractive rewards to those experiencing boredom or anxiety (62, 63). However, the social rewards and anxiety reduction effects provided by games are often temporary and may even increase social anxiety in real life (61). As a result, users may get caught in a vicious cycle of pathological gaming in an attempt to alleviate the increased social anxiety. In connection with this, a previous study found that children and adolescents with high scores in pathological gaming not only exhibited poorer quality of interpersonal relationships and more aggressive behavior but also had higher levels of anxiety compared to their peers (53). Moreover, in another study, adolescents with high scores in pathological gaming were found to experience more daily stress and exhibit higher levels of depression and anxiety (64). Additionally, in a separate longitudinal study involving 3,034 Singaporean children and adolescents, anxiety, along with self-control, was confirmed to have a significant impact on pathological gaming (65).

Loneliness is recognized as one of the key factors contributing to pathological gaming (66). Loneliness refers to the extent to which an individual experiences a deficiency and deterioration in both quantitative and qualitative aspects of relationships with others or society, it is a distressing and painful psychological experience that is exacerbated by the mismatch between expectations and the actual reality in interpersonal relationships (67).

Adolescents who lack social skills and experience loneliness may attempt to temporarily alleviate negative emotions stemming from social disconnection by forming relationships and participating in communities in the virtual world, it is raises concerns about an increased vulnerability to pathological (18). According to the ‘Social Compensation Hypothesis,’ the anonymous environment online can be seen as an opportunity for individuals experiencing social issues or those who are introverted to hide their identity or form new social relationships, potentially helping them forget the accumulated fatigue and fear associated with real-life social relationships (68, 69).

### 2.2 Social intelligence

Social intelligence refers to the ability to accurately understand oneself and others, perceive social situations, and manage and respond to social conflicts (70). In essence, social intelligence can be described as the ability to understand the social world well and act accordingly (71). Social intelligence is not only a crucial factor in collaboration and conflict resolution with others but is also closely related to an individual’s success, as it involves the ability to navigate social situations advantageously, particularly in social conflict scenarios (72, 73). At this juncture, the development of social intelligence can be influenced by negative psychological states such as loneliness, anxiety, and stress. Positive peer relationships and trust are known to be closely related to the mastery of social skills and the promotion of social intelligence (74, 75). Positive interpersonal relationships can themselves be conducive to enhancing social intelligence. Conversely, poor interpersonal relationships and loneliness can have a detrimental impact on the development of social intelligence as they are associated with a lack of opportunities for building effective social connections and fostering social cooperation.

Anxiety is also reported as a risk factor that can exacerbate deficits in social performance and impair social intelligence (76). For example, individuals experiencing social anxiety tend to overly self-monitor, evaluate their behavior more negatively, and have a higher likelihood of undervaluing themselves in social conflict situations (77). These tendencies can have an adverse impact on the smooth development of social intelligence. In line with this, several studies have reported results indicating that anxiety can have an unfavorable effect on social intelligence. In one study, individuals with social anxiety disorder were found to negatively evaluate their own social performance and achievements, leading to a greater likelihood of experiencing social rejection (78). Additionally, another study involving 110 German participants revealed a negative relationship between social anxiety and overall social intelligence (76).

Skills like social intelligence, which regulate emotional or social relationships, are essential for mitigating or recovering from stress (79). In other words, social intelligence can be considered a protective factor that alleviates the damage caused by stress. However, despite this, long-term accumulation of stress or high levels of overwhelming stress can continuously deplete psychological and social coping resources for handling problematic situations, resulting in impairments to cognitive, emotional, and perceptual functions and potentially leading to poor judgment in interpersonal relationships (17). Taking this into account, it is evident that long-term accumulated stress can have a detrimental effect on the development of social intelligence. In a study involving 309 university students, perceived stress was found to have a negative correlation with social information processing, social skills, and social awareness (41).

In general, high levels of social competence and social skills are known to act as protective factors that inhibit the occurrence of external relationship problems, thereby preventing pathological gaming behavior (18). On the other hand, low levels of social intelligence are considered one of the predictive factors for issues like pathological gaming. These findings are supported by research indicating that deficiencies in social skills and low levels of social intelligence can increase anxiety related to interpersonal relationship problems or serve as a means of escape from the pain associated with such problems, thereby facilitating pathological gaming behavior (70, 80–82). In essence, low social intelligence is one of the factors contributing to interpersonal relationship problems, and the resulting distress and fear can potentially serve as vulnerabilities that drive individuals to become excessively immersed in online games (21, 22). Conversely, high levels of sociability and strong interpersonal relationships are known as powerful protective factors that inhibit pathological gaming behavior (83). For instance, individuals with high levels of social intelligence are more likely to attempt problem resolution through interpersonal activities like negotiation when interpersonal conflicts arise, rather than resorting to games as a means of avoidance. In line with this, a study involving 582 middle and high school students in South Korea found that the group with pathological gaming behavior had relatively lower scores in social intelligence and social capital compared to the group without such behavior (10).

### 2.3 Aggression and pathological gaming

Aggression is a worldwide public health issue during adolescence, as its emotional, social, and economic consequences can have long-lasting and costly effects (84). Aggression refers to all intentional actions aimed at harming others, encompassing verbal aggression, physical aggression, as well as cognitive attributes such as hostility and emotional factors like anger (9, 85, 86).

On the other hand, aggression is known to be more significantly triggered by negative psychological states such as stress, anxiety, and loneliness. Psychological issues like anxiety can sometimes be accompanied by excessive aggression or conduct disorder, and particularly, high levels of chronic anxiety can act as a risk factor for the development of aggression in adolescents. For instance, damage to interpersonal relationships or crises can amplify an individual’s anxiety about their social reputation (87), as a result, abnormally high levels of anxiety can exacerbate emotional instability in the regulation system, leading to aggressive behavior (88). In line with this, research conducted on children and adolescents has found that individuals with higher levels of physical and verbal aggression tend to have higher anxiety scores (89).

Loneliness is also known as a negative internalized event that can trigger aggressive behavior (16). For instance, individuals with high levels of loneliness tend to perceive the intentions and actions of others more negatively in interpersonal relationships and may not seriously confront the causes of social (90). Especially when exposed to loneliness, individuals are prone to feeling negatively about not being accepted by others in situations of social conflict. As a result, they may try to control others’ reactions through relational aggression rather than resolving problems through dialogue and cooperation (91). In line with this, an online survey study conducted on 843 university students found that loneliness influences both aggressive behavior and smartphone addiction (67).

In order to protect the physical and mental health of adolescents, it is necessary to control the occurrence rate of aggressive behavior and its triggering factors and determinants. Various risk factors in each environment can trigger aggression in adolescents (92). High levels of stress can have a detrimental impact on both internalizing and externalizing problems in adolescents, including aggressive behavior (93). Aggression is one of the most important issues for adolescents in their relationships with others. For example, interpersonal stress such as rejection from peer groups can not only have developmental implications for adolescents but also induce the development of aggressive behavior (15). Generally, stress can activate the nervous system and induce negative moods, leading individuals to interpret neutral stimuli negatively (79). Adolescents, being in a stage of developmental rebellion, often exhibit high impulsivity in response to external stimuli. When faced with threats, they may find it difficult to regulate their emotions and often display various forms of aggressive behavior (94). Accumulated stress, in particular, can heighten sensitivity to aggression (95) and, by perpetuating negative psychological emotions and causing emotional dysregulation, make individuals more prone to aggressive responses to the same stimuli (93). In line with this, A study conducted on 1510 Spanish adolescents found a significant correlation between perceived stress and loneliness with aggressive (96).

Previous research has indicated that aggression is closely related to social and mental health issues, including loneliness, depression, impulsivity, and emotional regulation disorders (86, 97, 98). Additionally, aggression is known as a risk factor that contributes to problematic behaviors such as suicide and addiction (99–101), and it has been reported to have a strong association with pathological gaming behavior. Aggressive behavior not only harms the physical and mental health of adolescents but also affects their social, academic, and cognitive functioning (92). Nevertherless, some casual video games (102) or more purposefully designed serious video games have been proven to be effective in reducing symptoms of mental disorders such as depression and anxiety (103). So despite claims in some studies that video games can offer benefits across multiple domains, including cognitive, emotional, and social aspects (104). However, multiple studies have reported a high correlation between aggression and problematic gaming behavior (9, 19, 105). Furthermore, a survey study involving 424 university students found that both aggression and loneliness were identified as precursors to pathological gaming behavior (66). According to another related study, toxic behavior such as aggressive actions that can occur in competitive gaming environments may necessitate intervention programs to address them (106).

Furthermore, other studies suggest that individuals with higher levels of aggression may be more prone to pathological-immerse in video games with violent contexts when compared to those with lower levels of (105, 107). For example, aggressive and confrontational users may experience greater satisfaction when playing digital games that allow for aggression towards others. Popular game genres like Massively Multiplayer Online Role-Playing Games (MMORPGs) and First-Person Shooters (FPS) often require players to perform missions that involve attacking others to achieve victory (108). For adolescents with high levels of aggression, these games may serve as an attractive outlet for safely venting their built-up aggression, which may be restrained in real-life situations.

**Figure 1:**
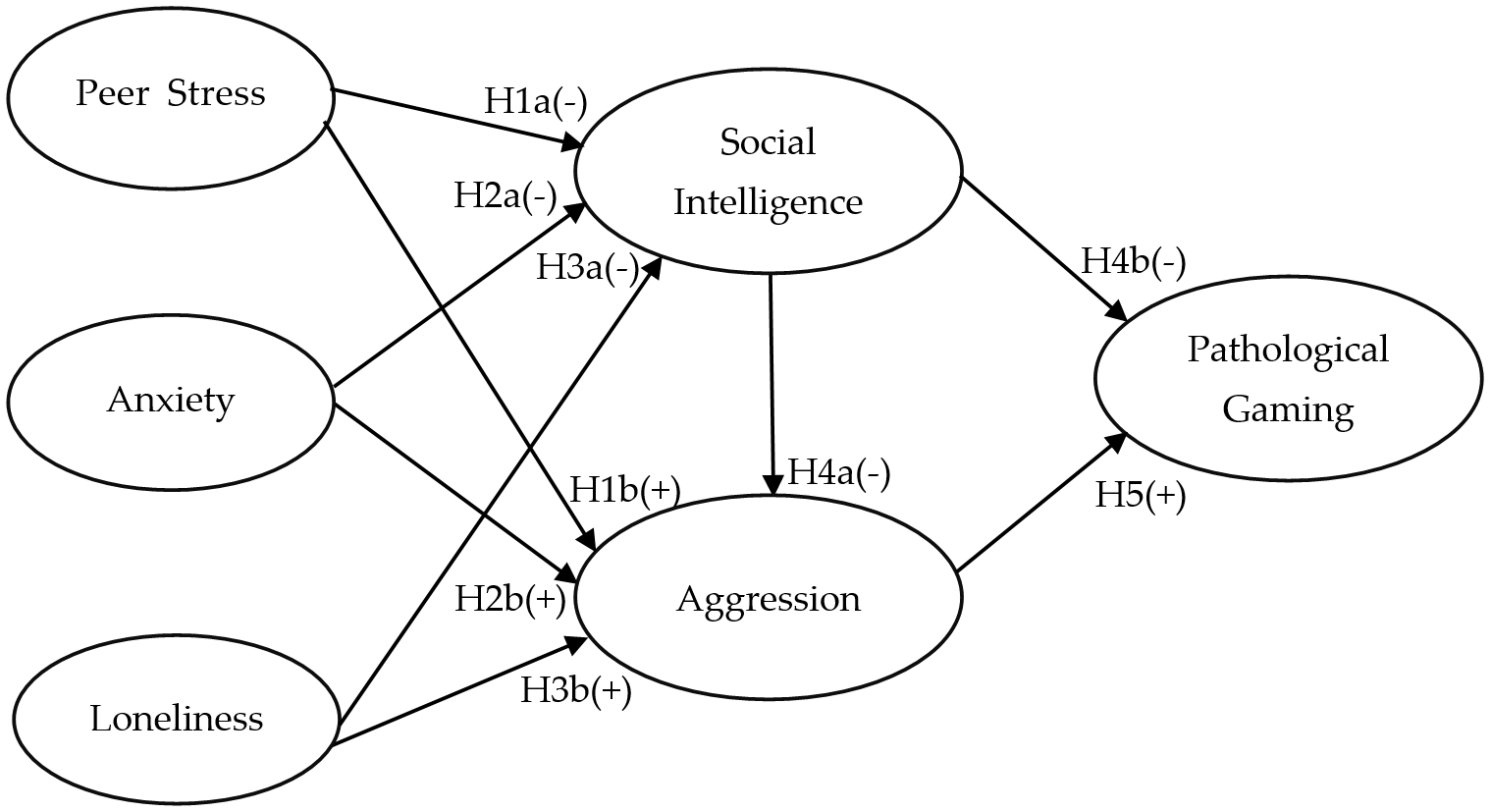
Research Model.

Based on previous studies, our research model (refer to **Figure 1**) demonstrates that adolescents’ negative emotions (peer stress, anxiety, loneliness) have the potential to influence social intelligence and aggression, with potential consequences for pathological gaming. These negative emotions may be regarded as contributing factors affecting both the social intelligence and aggression of adolescents, ultimately leading to the development of pathological gaming. To explore the influence of these social and emotional factors on pathological gaming, we posited the following hypotheses. We have formulated the following hypotheses.

H1. Peer Stress (PES) negatively influences Social Intelligence (SIT) (H1a) and positively influences Aggression (AGR) (H1b).
H2. Anxiety (AXT) negatively influences Social Intelligence (H2a) and positively influences Aggression (H2b).
H3. Loneliness (LON) negatively influences Social Intelligence (H3a) and is expected to have a significant impact on Aggression (H3b).
H4. Social Intelligence is expected to negatively influence Aggression (H4a) and negatively influence Pathological Gaming (PTG) (H4b).
H5. Aggression is expected to have a significant positive influence on Pathological Gaming.

## 3 Methods

### 3.1 Data collection

In this study, we utilized panel data from the Korean Adolescent Game User Cohort Research, conducted by the Korea Creative Content Agency (KOCCA) to assess the gaming behavior of primary, middle, and high school students, data collection took place from 2015 to 2018. The collection of panel data received prior approval from the ethics committee at Konkuk University, a collaborating institution. The survey process involved securing informed consent from respondents, ensuring the protection of their privacy and anonymity during data collection. A quota sampling approach, based on school grade and gender balance, was employed. Data were collected through 3 rounds of face-to-face interviews conducted by trained professionals at one year intervals, adhering to standardized survey protocols. The interviews adhered to established survey guidelines, maintaining consistency by employing the same questionnaire throughout the study. Participants received identical questionnaires throughout the entire study period and were remunerated with USD 27.00 each. Comprehensive details regarding the survey methodology and dataset can be found on the website (www.kocca.kr, accessed on Feb 20, 2024).

For the analysis of the Korea Creative Content Agency’s panel study on game users, a total of 968 Korean adolescents participated in the survey. Among them, there were 477 males (49.3%) and 491 females (50.7%). In terms of school levels, there were 345 elementary school students (35.6%), 333 middle school students (34.3%), and 290 high school students (30%). Students were questioned and responded regarding their gaming habits and their usual thoughts about gaming. **Table 1** below summarizes the demographic characteristics of the data participants.

**Table 1:** Demographic characteristics.

Characteristics		All Participants (968)	
		Frequency	(%)
Gender	Male	477	49.3
	Female	491	50.7
Age Group	Elementary Group	345	35.6
	Middle School	333	34.4
	High School	290	30
Online Game Duration (Daily Average)	Not Playing	100	10.3
	Under 30m	198	20.5
	30m ~ 1H	213	22
	1H ~ 2H	205	21.2
	2H ~ 3H	134	13.8
	3H ~ 4H	68	7
	4H ~ 5H	22	2.3
	5H ~ 6H	10	1
	Over 6H	18	1.9

### 3.2 Measurement

A structural equation modeling (SEM) and repeated measures analysis, which uses the GLM (General Linear Model), were used to verify the research questions. The questionnaire included items measuring constructs such as stress, anxiety symptoms, loneliness, as well as social intelligence and aggression. Literature adopted for the questionnaire was typically sourced from validated measures used by previous researchers. Various Likert scales were employed for item-level measurement of each construct. However, a Likert scale was not used for measuring gaming time.

#### 3.2.1 Peer stress

In order to measure peer stress, three items pertaining to interpersonal relationships were selected from the Life Stress Scale (109). The scale consisted of 3 items, and responses were structured using a 3-point Likert scale (3=frequent, 2=average, 1=not at all). For example, items such as “I couldn’t have a conversation with friends” and “I couldn’t make friends who I could relate to” were included (α = 0.757).

#### 3.2.2 Anxiety

To measure anxiety, we employed the GAD (Generalized Anxiety Disorder) scale (110). The anxiety measurement comprised 7 items, with responses using a 4-point Likert scale (3=most of the time - nearly every day, 2=sometimes - more than once a week, 1=occasionally - a few days, 0=never). For instance, sentences like “Found it difficult to relax,” “Felt anxious and worried,” and “Experienced extreme restlessness” were included (α = 0.890).

#### 3.2.3 Loneliness

To measure the degree of loneliness, we utilized the UCLA Loneliness Scale. The loneliness measurement included 10 items, with responses structured using a 4-point Likert scale (4=very much, 3=somewhat, 2=hardly, 1=not at all). For instance, sentences like “Feel lonely” and “Experience a sense of isolation from others” were included (α = 0.912).

#### 3.2.4 Social intelligence

To measure social intelligence, the Tromso-Social Intelligence Scale was employed. The social Intelligence measurement included 21 items, with responses structured using a 5-point Likert scale (5=very much, 4=quite, 3=average, 2=no, 1=not at all). The subscales of the items consisted of seven questions each, focusing on Social Information Processing, Social Skill, and Social Awareness. Items included statements like “I can predict the behavior of others” and “I am often surprised by the unexpected reactions of others to my actions” (α = 0.859).

#### 3.2.5 Aggression

The Short-Form Buss–Perry Aggression Questionnaire (BPAQ-SF) was utilized to assess adolescent aggression. Bryant and Smith condensed the original 29-item aggression scale, the Buss–Perry Aggression Questionnaire developed by Buss and Perry, to a 12-item version. The responses were structured using a 5-point Likert scale (5=very much, 4=mostly, 3=occasionally, 2=mostly not, 1=not at all). For example, statements such as “I frequently have disagreements with others” and “Sometimes I get angry for no apparent reason” were included (α = 0.880).

#### 3.2.6 Pathological gaming

For measuring the extent of pathological gaming in adolescents, a modified version of the established Internet Addiction Scale (111) was utilized to better accommodate the gaming context. The pathological gaming measurement included 20 items, and responses were structured using a 5-point Likert scale (5=very much, 4=mostly, 3=occasionally, 2=mostly not, 1=not at all). This place includes questions such as “I’ve had occasions where I couldn’t sleep because I stayed up late playing games” and “I have had times when, not playing games, my mind was preoccupied with imagining playing games or thinking about gaming” were included (α = 0.940).

## 4 Results

### 4.1 Reliability and Validity test

We measured the levels of peer stress, loneliness, and anxiety in 968 adolescents (T1) and the levels of social intelligence (T2), aggression (T2), and pathological gaming (T3) in these adolescents. Between T1, T2, and T3, there exists an interval of 1 year each. We conducted reliability, correlation, and validity testing on the measurement values obtained through the tests (refer to **Table 2**). The reliability tests included Cronbach’s alpha, composite reliability (CR), and average variance extracted (AVE). The results of these tests indicated that the scores were all valid for the model (with a CR of 0.7 and an AVE of 0.5). In the case of missing data, we used the regression imputation method provided by the Amos program. This method replaces missing data with imputed values based on linear regression analysis between variables.

**Table 2:** Results for Measurement Model.

Scale/Items	Cronbach’ α	M	SD	CR	AVE	R2
Peer Stress (PES)	0.757	0.67	0.555	0.763	0.673	
Anxiety (AXT)	0.89	0.46	0.543	0.894	0.645	
Loneliness (LON)	0.912	1.56	0.533	0.918	0.618	
Social Intelligence (SIT)	0.859	5.02	1.165	0.864	0.703	0.098
Aggression (AGR)	0.88	1.8	0.773	0.886	0.627	0.386
Pathological Gaming (PTG)	0.94	2.22	0.976	0.943	0.705	0.236

M, Mean; SD, Standard Deviation; CR, Composite Reliability; AVE, Average Variance Extracted; R2, R Square Adjusted.

### 4.2 Research model test

The data were analyzed utilizing the PLS-SEM method. Within the framework of PLS-SEM statistical processing, the measurement model is assessed through statistical criteria including convergent validity (such as factor loading values, AVE), internal consistency reliability (e.g., Cronbach’s alpha value, CR), and discriminant validity. We conducted an HTMT analysis to assess the discriminant validity among latent variables in the structural equation model for our research (refer to **Table 3**). The results confirmed the validity and appropriateness of all indices between variables, thus allowing for discriminant validity.

**Table 3:** Heterotrait-Monotrait Ratio (HTMT) for Discriminant Validity.

Variables	PTG	LON	AGR	AXT	SIT	PES
Pathological Gaming (PTG)						
Loneliness (LON)	0.313					
Aggression (AGR)	0.366	0.381				
Anxiety (AXT)	0.251	0.466	0.397			
Social Intelligence (SIT)	0.331	0.297	0.644	0.288		
Peer Stress (PES)	0.236	0.592	0.372	0.407	0.265	

Shaded boxes are the standard reporting format of PLS-SEM HTMT analysis.

**Figure 2:**
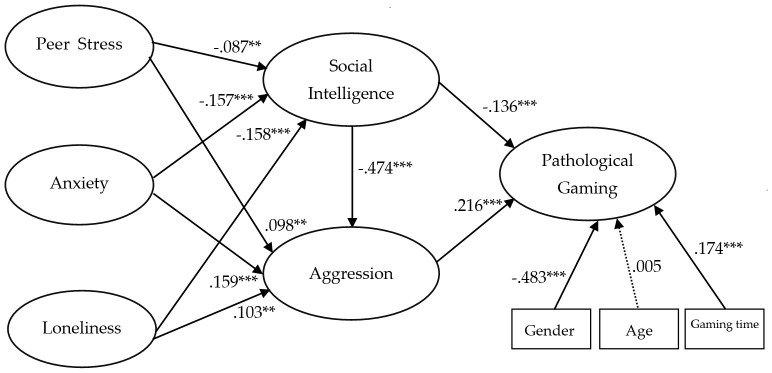
Research model and hypothesis.

Based on the evaluation of the measurement model (refer to **Table 4**), we tested our hypotheses through the analysis of the structural model (refer to **Figure 2**). All hypotheses were statistically supported in the structural model analysis, and the hypothesis testing results are summarized as follows.

**Table 4:** Results of the hypothesis tests.

Hypothesis	Coef.	Mean	SD	t	Results
H1a. Peer stressors (PES) → Social intelligence (SIT)	-0.087	-0.089	0.034	2.591**	Accepted
H1b. Peer stressors (PES) → Aggression (AGR)	0.098	0.099	0.035	2.836**	Accepted
H2a. Anxiety (AXT) → Social intelligence (SIT)	-0.157	-0.159	0.034	4.624***	Accepted
H2b. Anxiety (AXT) → Aggression (AGR)	0.159	0.16	0.033	4.859***	Accepted
H3a. Loneliness (LON) → Social intelligence (SIT)	-0.158	-0.157	0.037	4.324***	Accepted
H3b. Loneliness (LON) → Aggression (AGR)	0.103	0.104	0.033	3.156**	Accepted
H4a. Social intelligence (SIT) → Aggression (AGR)	-0.474	-0.472	0.026	17.992***	Accepted
H4b. Social intelligence (SIT) → Pathological gaming (PTG)	-0.136	-0.135	0.035	3.887***	Accepted
H5. Aggression (AGR) → Pathological gaming (PTG)	0.216	0.217	0.037	5.886***	Accepted
[Control variable] Gender → Pathological gaming (PTG)	-0.483	-0.485	0.06	8.008***	–
[Control variable] Age → Pathological gaming (PTG)	0.005	0.005	0.029	0.19	–
[Control variable] Online game duration → Pathological gaming (PTG)	0.174	0.176	0.03	5.729***	–

Coef., Coefficient; Significant level: ** p < 0.01, *** p <.001.

One of the psychological factors, peer stress (T1), was found to have a significant negative impact on social intelligence (T2) (β=-0.087, p<0.01) and had a significant impact on aggression (T2) (β=0.098, p<0.01). In the relationship between anxiety symptoms (T1) and social intelligence (T2), anxiety symptoms were found to have a significant negative impact on social intelligence (β=-0.157, p<0.001) and had a significant impact on aggression (T2) (β=0.159, p<0.001). In the relationship between loneliness (T1) and social intelligence (T2), loneliness was found to have a significant impact on social intelligence (β=0.159, p<0.001) and had a significant impact on aggression (T2) (β=-0.103, p<0.01).

In the relationship between social intelligence (T2) and aggression (T2) and pathological gaming (T3), social intelligence was found to have a significant negative impact on aggression (β=-0.474, p<0.001) and had a significant negative impact on pathological gaming (T3) (β=-0.136, p<0.001). In the relationship between aggression (T2) and pathological gaming (T3), aggression had a significant impact on pathological gaming (β=0.216, p<0.001). Therefore, adolescent peer stress, anxiety, and loneliness affect social intelligence and aggression, and social intelligence and aggression were found to have a significant impact on pathological gaming.

Our hypothesis testing results were consistent with the expectations of the research group. Psychological factors such as Peer Stress, Anxiety Symptoms, and Loneliness were found to have a negative impact on Social Intelligence, with Loneliness having the largest negative impact. Anxiety Symptoms also significantly affected Social Intelligence, with a slight difference in the degree of negative impact compared to Loneliness. Social Intelligence had a very significant negative impact (β=-0.474, p<0.001) on Aggression and a negative impact (β=-0.136, p<0.001) on Pathological Gaming. Aggression was found to have a significant positive impact on Pathological Gaming. Therefore, in the context of adolescent gaming, Social Intelligence emerged as a key variable that significantly influences Aggression and can lead to Pathological Gaming.

## 5 Discussion

### 5.1 Findings

This study focused on exploring the factors that induce or inhibit pathological gaming in adolescents, with a particular emphasis on investigating the association between these factors. The study examined how psychological factors affect social intelligence and aggression in adolescents and how this, in turn, influences pathological gaming. The research model placed the focus on whether social intelligence is associated with negative emotions and if it, in turn, has a significant associate with aggression or pathological gaming.

First, psychological factors such as peer stress, anxiety, and loneliness were found to have a negative association with social intelligence. Among these, loneliness exhibited the most significant negative association with social intelligence. Anxiety symptoms also showed a substantial negative association with social intelligence, with a minor difference in the degree of negative association compared to loneliness. These results suggest that the awareness of being isolated from peers and psychological instability are potent negative emotional factors that inhibit the development of social intelligence. This implies that for the smooth development of social intelligence, it is necessary to consider not only network factors like the size of interpersonal relationships but also the mental health of adolescents.

The results of our hypothesis testing aligned with the expectations of the research group. According to previous research, high levels of negative emotions can lead to self-isolation or problems in smooth communication, and in severe cases, can adversely affect social intelligence by increasing negative evaluations of social performance and achievements or by intensifying compulsive self-monitoring (41, 77). Meanwhile, it has been found that peer stress negatively impacts social intelligence, but the magnitude of its impact is relatively less than that of anxiety or loneliness. These results indicate the necessity to consider social intelligence generally as a protective factor contributing to the mitigation and recovery from stress (79). However, intense interpersonal relationship stress can deplete and impair cognitive, emotional, and perceptual functions and resources needed for coping with social problems, potentially undermining its function as a protective factor. In this context, it can be interpreted that while peer stress has a lesser negative impact on social intelligence compared to loneliness and anxiety, it can still be threatening in high-intensity situations.

Furthermore, this study has found a significant negative correlation between social intelligence and aggression. This indicates that adolescents with a higher level of social intelligence are less likely to possess hostile and aggressive behavior or intentions towards others. Some previous research suggests that social intelligence can influence aggression and conflict behavior through various pathways (112). For instance, social intelligence may increase the likelihood of an individual adopting peaceful means in social conflicts when there is a lack of control over empathy. This is because it is most efficient and less risky for people with high social intelligence to choose ways to expose themselves as little as possible to interpersonal crises. However, the aforementioned studies also concurrently point out that social intelligence is associated with indirect aggression. Therefore, the findings of this study indicating that social intelligence negatively impacts aggression align partially with previous research. Yet, it also suggests that interpretations should be approached cautiously, as differentiating the forms of aggression could potentially yield varied results.

Both social intelligence and aggression have been shown to significantly influence pathological gaming. Social intelligence appears to have a positive impact on pathological gaming, whereas aggression positively influences pathological gaming. These findings are consistent with previous research on pathological gaming (21, 22, 105, 107). For example, adolescents with high levels of aggression may exhibit pathological gaming behaviors as a means to release suppressed aggressive emotions and derive pleasure, particularly through the use of violent games. Conversely, a high level of social intelligence seems to influence the prevention of interpersonal problems, thereby inhibiting escapist gaming behaviors or excessive gaming for social reputation management (83). These results support the notion that mitigating aggression and fostering social intelligence in adolescents are necessary to curb pathological gaming behaviors.

This longitudinal study presented in this paper supports the notion that adolescents’ initial negative emotions are involved in the development of psychosocial traits and competencies, such as social intelligence and aggression, which ultimately can impact pathological behavior. Particularly significant is the finding that negative emotions like anxiety, stress, and loneliness contribute to pathological gaming through specific psychosocial factors. Unearthing and analyzing the hidden relationship between negative emotions and pathological gaming is a crucial condition for developing effective and practical preventive measures for youth mental health issues. Considering that previous research on pathological gaming mainly focused on key psychological variables, including self-control, the discovery of the impact of social intelligence in this study is especially important. In adolescence, a period when relationships with peers and the performance of social tasks become increasingly significant, social intelligence has a crucial impact on the recognition and induction of problematic behaviors (83). Therefore, it is essential to understand through longitudinal data what factors form or inhibit the development of adolescents’ social intelligence. In this context, the findings of this study, which longitudinally examined the pathway from negative emotions to pathological gaming, including the impact of aggression and social intelligence, underscore the importance of addressing early negative emotions and major psychosocial traits. This approach suggests that social and health-related attention to these aspects can aid in resolving issues of media over-engagement among adolescents.

### 5.2 Theoretical and practical implications

The answers regarding the association between adolescents’ social intelligence and aggression and their impact on pathological gaming align with the results of this study and are consistent with previous research findings. The emergence of negative emotions and issues in social relationships is one of the key predictors of pathological gaming, and it can trigger dynamic relationships among various psychological variables such as stress, anxiety, loneliness, aggression, and social intelligence, as advocated by previous studies (19, 20, 28, 36). Through the hypotheses in this study, we have confirmed a significant association between social intelligence and aggression’s impact on pathological gaming. Social intelligence exhibits a strong association with aggression, and as aggression levels increase, the likelihood of pathological gaming also rises. This intuitive model provides a clear and straightforward explanation of how social intelligence and aggression can interact organically to affect pathological gaming.

The comprehensive results of this study highlight some key points. First, a new finding that social intelligence can have a much more significant association with adolescent aggression than we initially expected suggests that by regulating adolescents’ social abilities, we can prevent pathological gaming. Second, taking a holistic view from the perspective of adolescent gaming, negative emotions that adolescents may experience during their adolescent years are strongly associated with social intelligence, with social intelligence having a stronger association with aggression than negative emotions. Furthermore, this aggression that is formed in such a manner also exhibits a significant association with pathological gaming. What sets our research findings apart is the discovery that we can address aggression through adolescents’ social intelligence. Adolescence is a period in which various conflicts and difficulties can arise, as it includes the process of individuals establishing their values, beliefs, future visions, and shaping their identity in terms of social roles, among other aspects. From the perspective of previous research (107), that factors in the adolescent environment can affect their stress and specific pathological behaviors, the idea that social intelligence influences aggression as much as it does provides an opportunity to enhance adolescent well-being by preventing the induction of negative emotions through care and support in their surrounding environment.

Notable, this study discovered a new mediating factor in the relationship between negative emotions arising from the adolescent environment, such as peer stress, anxiety, loneliness, and aggression, and pathological gaming, which is social intelligence. To the best of our knowledge, this is the first longitudinal attempt to explore these relationships. Despite the growing body of research on adolescent pathological gaming, studies focusing on factors related to social intelligence are scarce.

Prior research has primarily emphasized the role of negative emotions like stress, anxiety, and loneliness in triggering pathological gaming. However, our study extends the implications of the existing findings that suggest a connection between negative emotions and adolescent pathological gaming. By shifting the focus to the management of the new factor, social intelligence, and its role in reducing aggression, this study offers new guidelines for addressing pathological gaming. Thus, in-depth analyses regarding the role of social intelligence require ongoing discussion.

Furthermore, this study provides significant insights for the development of educational and healthcare policies and systems aimed at adolescent mental health. As demonstrated by the results of this study, pathological gaming is likely to be triggered by negative emotions and psychosocial competencies, with aggression having a greater influence than gaming duration. These findings underscore the importance of preventing adolescents from being overwhelmed by negative emotions and, even when exposed to such emotions, guiding them away from developing negative traits like increased aggression. For instance, enhancing healthcare counseling support systems to ensure negative emotions are not prolonged and are adequately addressed, or creating cultural and educational environments, could be viable solutions. Additionally, considering the impact of social competencies, including social intelligence, on pathological gaming, it would be beneficial to provide separate support for adolescents with underdeveloped interpersonal skills to prevent them from resolving anxiety and fear in inappropriate ways.

However, despite the empirical results we have presented, this study has the following limitations. Firstly, the use of panel data is limited in generalizing our research model. Since the data was collected from Korean adolescents, different results may be obtained in other countries with different cultures. Secondly, the “social intelligence” assessed in this study was self-determined by the adolescents who were the subjects of the research through a questionnaire. Consequently, there might be a slight difference, given the possibility that subjective interpretations about oneself could minimally influence self-evaluation during adolescence. Therefore, in future research, measuring the actual social intelligence of adolescents using a more systematic approach could enhance the validity of the research findings. In future research, it will be necessary to use a more diverse range of psychosocial variables related to social intelligence. For instance, social intelligence might be related to psychological variables associated with interpersonal relationships and social competence, such as self-esteem. Additionally, this study did not further examine the relationships between variables, including latent variables. Therefore, more in-depth analyses will be required in subsequent research.

## Data availability statement

Publicly available datasets were analyzed in this study. This data can be found here: https://www.kocca.kr/gameguide/subPage.do?menuNo=203709.

## Ethics statement

The studies involving humans were approved by Konkuk University Institutional Review Board. The studies were conducted in accordance with the local legislation and institutional requirements. Written informed consent for participation in this study was provided by the participants’ legal guardians/next of kin.

## Author contributions

SL: Conceptualization, Investigation, Writing – original draft. EJ: Project administration, Supervision, Validation, Writing – review & editing. JC: Data curation, Investigation, Methodology, Writing – original draft. MP: Formal analysis, Investigation, Methodology, Writing – original draft.
